# A Modular Vaccine Development Platform Based on Sortase-Mediated Site-Specific Tagging of Antigens onto Virus-Like Particles

**DOI:** 10.1038/srep25741

**Published:** 2016-05-12

**Authors:** Shubing Tang, Baoqin Xuan, Xiaohua Ye, Zhong Huang, Zhikang Qian

**Affiliations:** 1 Unit of Herpesvirus and Molecular Virology, Key Laboratory of Molecular Virology & Immunology, Institut Pasteur of Shanghai, Chinese Academy of Sciences, University of the Chinese Academy of Sciences, Shanghai 200031, China; 2 Unit of Vaccinology and Antiviral Strategies, Key Laboratory of Molecular Virology & Immunology, Institut Pasteur of Shanghai, Chinese Academy of Sciences, University of the Chinese Academy of Sciences, Shanghai 200031, China

## Abstract

Virus-like particles (VLPs) can be used as powerful nanoscale weapons to fight against virus infection. In addition to direct use as vaccines, VLPs have been extensively exploited as platforms on which to display foreign antigens for prophylactic vaccination and immunotherapeutic treatment. Unfortunately, fabrication of new chimeric VLP vaccines in a versatile, site-specific and highly efficient manner is beyond the capability of traditional VLP vaccine design approaches, genetic insertion and chemical conjugation. In this study, we described a greatly improved VLP display strategy by chemoenzymatic site-specific tailoring antigens on VLPs surface with high efficiency. Through the transpeptidation mediated by sortase A, one protein and two epitopes containing N-terminal oligoglycine were conjugated to the LPET motif on the surface of hepatitis B virus core protein (HBc) VLPs with high density. All of the new chimeric VLPs induced strong specific IgG responses. Furthermore, the chimeric VLPs with sortase A tagged enterovirus 71 (EV71) SP70 epitope could elicit effective antibodies against EV71 lethal challenging as well as the genetic insertion chimeric VLPs. The sortase A mediated chemoenzymatic site-specific tailoring of the HBc VLP approach shows great potential in new VLP vaccine design for its simplicity, site specificity, high efficiency, and versatility.

As natural nanoparticles, VLPs are widely applied in nanotechnologies, including bioimaging^1^, drug delivery^2^, and electronics^3^. VLPs are used most often in vaccinology. VLPs trigger strong humoral and cellular immune responses by mimicking infection with virions^4^. VLPs are safer than traditional vaccines made of inactivated or attenuated viruses because they are devoid of infectious genomes^5^. VLPs are nanoscale particles that are favorably taken up by antigen-presenting cells (APCs) to facilitate B- and T-cell responses. The repetitive multivalent display of antigens on the VLP surface in high density cross-link B-cell receptors so as to stimulate a strong antibody response^6^. VLPs have been extensively developed as delivery carriers to display foreign antigens for prophylaxis against virus infection^7^. In addition, antigen-based VLPs show promising potential in therapeutic treatment of cancer, nicotine addiction, and other diseases^8^.

Displaying antigens on the surface of VLPs is of great importance for applications in prophylactic and immunotherapeutic vaccination. There are currently two classic chimerical vaccine design approaches based on VLPs, genetic insertion and chemical conjugation^9^. Genetic insertion is easily achieved by genetic engineering, and antigens are presented on VLPs surface with high efficiency. However, on occasion, genetic fusion chimeric proteins form inclusion bodies instead of VLPs^10^. It also takes weeks to fabricate a new vaccine from genetic engineering to vaccine production, which is inconvenient for new vaccine manufacture^11^. This limit is not a problem in chemical conjugation because of its convenience and versatility in coupling different antigens on to VLPs. Chemical conjugation includes non-covalent conjugation and covalent conjugation. Non-covalently attached antigens on the VLP surface sometimes failed to induce an immune response^12^. Several covalent chemical conjugation methods have been developed. Despite its rather high efficiency, the Cu(I) catalyzing azide and alkyne click chemistry to couple proteins on VLP surfaces reduces protein bioactivity by 3- to 5-fold^13^. Another common chemical conjugation method is to activate lysine on the VLP surface with a heterobifunctional cross-linker such as maleimidobenzoic acid sulfosuccinimidyl ester (Sulfo-MBS), which will ligate with free cysteine. The coupling efficiency of proteins^14^,^15^ and peptides^16^,^17^ that contain cysteine at the C-terminal to the VLP surface is usually less than 40%. A great excess of bifunctional crosslinker is needed. Peptides that contain cysteine or protein surfaces exposed with cysteine lead to improper orientation, which limits the application of this method. A more specific chemical conjugation method was developed by aniline-catalyzed oximation ligation with coupling efficiency around 50%^12^. However, this covalent ligation is rather complicated and involves the use of multiple reaction processes and extraordinary excess linkers. In summary, the existing approaches are not satisfactory to display different antigens on VLPs.

In this study, we developed a simple and site-specific vaccine design approach with high efficiency. Antigens are simply covalently linked to HBc VLPs by chemoenzymatic site-specific coupling mediated by sortase A, sortagging for short, in one reaction. The HBc VLP platform, one of the most advanced VLP display platforms, is used as a scaffold^18^. The exposed LPETGG motif on the surface of the VLPs is recognized by sortase A, and N-terminal oligoglycine antigens are coupled to the conserved motif by transpeptidation mediated by sortase A^19^. We attempted to ligate the AD-4 domain on glycoprotein B (gB) of human cytomegalovirus (HCMV) and two defined linear eptitopes of enterovirus 71 (EV71) to N-LPETGG-C VLPs under transpeptidation by sortase A. In a previous study, human antibodies binding AD-4-GST fusion protein expressed in *E. coli* exhibited a high neutralizing capacity against HCMV^20^. Their results indicated that AD-4 was a potential vaccine design target for HCMV. We expressed GGGS-AD-4-6xHis (GGGS-AD-4 for short) and coupled it to N-LPETGG-C VLPs with more than 70% efficiency. The chimeric VLPs elicited a much stronger and faster anti–GGGS-AD-4 IgG response than protein. However, the new chimeric VLPs induced antibodies that showed no inhibition of HCMV entry. Our results suggested that AD-4 itself was not an effective subunit vaccine against HCMV. GGG-SP55 and GGG-SP70^21^, two defined epitopes of EV71, were conjugated to N-LPETGG-C VLPs with more than 90% efficiency. All of the antibodies triggered by VLPs sortagged with EV71 epitopes and VLPs fused to EV71 epitopes were shown to block EV71 entry. The anti-SP70-based antibodies induced by genetic insertion and sortagging provided approximately equal protective efficacy against lethal EV71 challenge.

Through sortase A mediated tagging, a protein domain and two epitopes are conjugated to VLPs surface with high density. All of the chimeric VLPs induced a strong and specific IgG response. Our results demonstrate that our platform is versatile and convenient for coupling of different antigens, which is beyond the capacity of genetic insertion. In addition, our platform is simpler, more efficient, and more site specific than chemical conjugation. In general, the novel vaccine design strategy proposed in our study surpasses traditional approaches and can be used to tailor VLP surfaces for further applications.

## Results

### Design of sortase-mediated coupling system for site-specific linking of antigens to HBc VLPs

**Figure 1:**
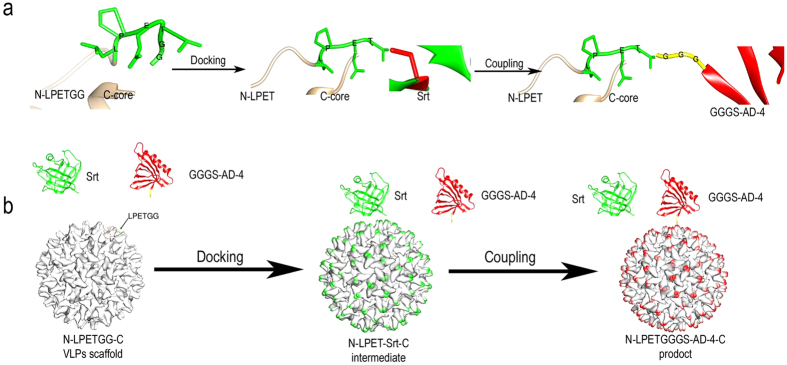
Schematic depiction of chemoenzymatic site-specific coupling of GGGS-AD-4 to N-LPETGG-C VLPs. (**a**) Site-specific focus view of the 3D structures at the LPETGG site during coupling process by UCSF Chimera. The LPETG motif on the N-LPETGG-C VLP surface is recognized by sorase A-4, and the T- and G-bond is cleaved by enzyme. The sortase A-4 is acylated at cysteine 184 with threonine at the “docking” step. The intermediate is then resolved by GGGS-AD-4 nucleophilic attack. GGG-based antigens are thus coupled to the VLP scaffold. (**b**) The dynamic process of reaction is illustrated. The models of sortase A-4 (Srt, green), GGGS-AD-4 (red), and N-LPETGG-C VLPs (white) were generated by MODELLER and viewed with UCSF Chimera. At the docking step, sortase A-4 forms an intermediate with the VLP scaffold, and the spikes of the VLPs are decorated by sortase A-4 (green). GGGS-AD-4 then couples to the VLP, and the spikes are modified by GGGS-AD-4 (red).

We designed a strategy for efficient and versatile coupling of foreign antigens to HBc VLPs in a site-specific manner (Fig. 1). In this strategy, split HBc VLPs are used as a scaffold. HBc is split into two parts, amino acid (aa) 1-79 N terminal (N-core) and aa 80-183 C terminal (C-core), at the immunodominant c/e1 loop^22^. The conserved LPETGG motif is fused to the C terminal of the N-core (named N-LPETGG) to be exposed on the HBc VLPs surface, which is recognized by the transpeptidase, sortase A. N-LPETGG and C-core still form a VLPs structure (instructed as N-LPETGG-C). Sortase A breaks the bond between threonine and glycine residues, and the cysteine 184 of sortase A is linked to N-LPET. An acylated intermediate is formed at the “docking” step. Sortase A is then released from the VLP scaffold by peptides or proteins that harbor the N-terminal oligoglycines nucleophilic attack^23^. Hence, oligoglycine-based antigens are tagged on the N-LPETGG-C VLP surface at the “coupling” step. After docking and coupling, N-terminal oligoglycine-based antigens are conjugated to the surface of the N-LPETGG-C VLPs. N-terminal truncation of 59 aa of sortase A was used in our study^24^ with four site mutations (sortase A-4 for short) to improve enzyme activity^25^.

### Generation of components of the coupling system

**Figure 2:**
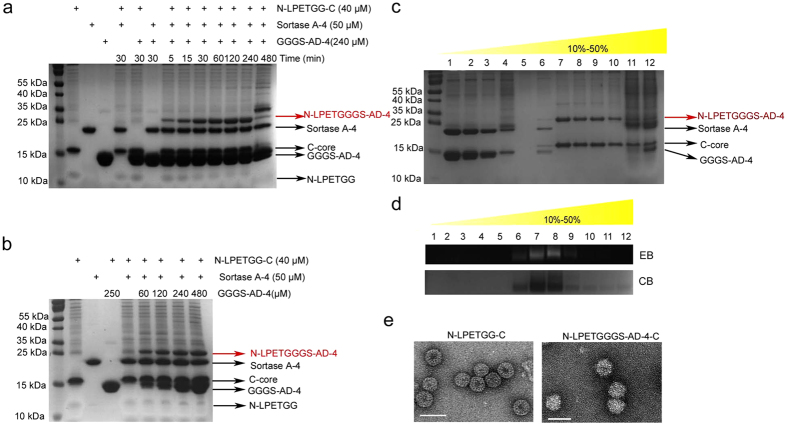
Chemoenzymatic coupling of GGGS-AD-4 to N-LPETGG-C VLPs without impairing the VLP structure. (**a**) Time-dependent assay of sortagging GGGS-AD-4 to VLPs. Sortagging reached a balance after 1 hour of incubation. Target product was labeled with a red arrow. Other components used in coupling system were instructed in black arrows. (**b**) Optimization of the concentrations of the substrate. No apparent increase in the target product was observed when the substrate was more than 3-fold. (**c**) Tricine-SDS-PAGE analysis and (**d**) NAGE analysis were performed to monitor the sucrose gradient density ultracentrifugation separation of the target product. NAGE was stained with ethidium bromide (EB) and coomassie brilliant blue (CB). (**e**) VLP structures were confirmed by transmission electron microscopy (TEM). White bar: 50 nm.

The components used in the coupling system are described in Methods. In brief, N-LPETGG-C VLPs were sequentially purified by ammonium sulfate precipitation and sucrose gradient density ultracentrifugation with slight modification of the ultracentrifugation conditions according to a previous report^26^. The 180 copies (T3) or 240 copies (T4) of N-LPETGG-C formed VLPs that were mainly distributed among fractions 6 to 10 according to Tricine-SDS-PAGE^27^ and native agarose gel electrophoresis (NAGE)^26^ staining assay (Supplementary Fig. 1). According to our results, we found that the N-LPETGG protein was not preferred by coomassie blue staining (Supplementary Fig. 1a). The purified VLP by sucrose gradient centrifugation should have equal molar of N-LPETGG and C-core, yet the staining for N-LPETGG was still much weaker (Fig. 2a,b). This phenomenon also existed in a previous study^22^. The VLP structure was confirmed by transmission electron microscopy (TEM) (Fig. 2e). Transpeptidase (Supplementary Fig. 2) and GGGS-AD-4 (Supplementary Fig. 3) were expressed from pET28a in *E. coli* and purified by immobility metal affinity chromatography (IMAC) and anion exchange. The aggregates of sortase A-4 were removed by sucrose gradient density ultracentrifugation (Supplementary Fig. 2c).

### Sortagging GGGS-AD-4 to VLPs surface with high density

Transpeptidation was accomplished at room temperature with conditions similar to the previous study^28^. To evaluate the kinetics of sortagging, we carried out a time-dependent assay. The reversible sortagging reached a balance after 1 hour of incubation (Fig. 2a). Excess incubation resulted in unexpected byproducts. To optimize the concentrations of antigens, we tested different folds of GGGS-AD-4. Three fold of GGGS-AD-4 resulted in around 70% of VLPs occupied by the interested protein. Further increase the amount of GGGS-AD-4 to 6 fold molar ratio slightly enhanced the sortagging efficiency (Fig. 2b). We thus chose six fold of GGGS-AD-4 in sortagging to balance the coupling efficiency and substrate concentration. The stoichiometric ratio of the reaction components was 1 fold N-LPETGG-C VLPs, 1.25 fold sortase A-4, and 6 fold GGGS-AD-4. The coupled products were separated by sucrose gradient density ultracentrifugation with a recovery more than 50%. The conjugated products were distributed in a density similar to that of the N-LPETGGG-C VLPs according to Tricine-SDS-PAGE analysis (Fig. 2c) and NAGE staining assay (Fig. 2d). The intact VLP structure was observed by TEM (Fig. 2e).

### Enhanced immunogenicity of GGGS-AD-4 coupled onto VLPs

**Figure 3:**
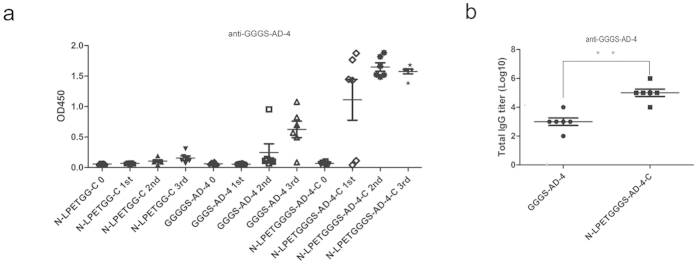
N-LPETGG-C VLPs coupled with GGGS-AD-4 elicited a much stronger specific IgG response than GGGS-AD-4. (**a**) Immunization of N-LPETGGGS-AD-4-C VLPs significantly improved the specific IgG response compared to that of GGGS-AD-4. Anti–GGGS-AD-4 antibodies were faster and stronger when induced by N-LPETGGGS-AD-4-C VLPs than when induced by GGGS-AD-4. Blood samples before antigens injection (named 0), first time injection (1st), second and third time booster (2nd and 3rd) were analyzed by ELISA. (**b**) Anti-GGGS-AD-4 IgG response was 100 fold increased after coupling GGGS-AD-4 to N-LPETGG-C VLP scaffold (**P < 0.001, by two-tailed student’s t-test).

GGGS-AD-4 protein and N-LPETGG-C VLPs sortagged with GGGS-AD-4 (N-LPETGGGS-AD-4-C) were immunized to mice with PBS and N-LPETGG-C VLPs as control. All six mice in each group received the first intraperitoneal injection at week 0 and boosters at weeks 2 and 4. Serum samples were collected 2 weeks after each immunization. Only GGGS-AD-4 and N-LPETGGGS-AD-4-C induced an anti–GGGS-AD-4 IgG response. After the first injection, GGGS-AD-4 sortagged to VLPs stimulated a greater average specific IgG response than the third booster of GGGS-AD-4. Second booster of N-LPETGGGS-AD-4-C elicited very strong specific IgG response and further booster would not enhance IgG response (Fig. 3a). Then we measured the titers of anti-GGGS-AD-4 IgG antibodies, and found that that specific IgG response was increased by 100 fold after coupling GGGS-AD-4 to VLP scaffold (Fig. 3b). The immunogenicity of GGGS-AD-4 was greatly improved after sortagging to N-LPETGG-C VLPs. However, neutralizing analysis against the AD169 strain of HCMV showed that anti–GGGS-AD-4 IgG antibodies had no effect in blocking HCMV entry (data not shown). Through B-cell repertoire analysis, the discontinuous AD-4 domain was identified as the binding target of the most effectively neutralized antibodies^20^. Binding of viruses with a tyrosine (Y) at position 364 and a lysine (K) at position 379 mutations with anti–AD-4 antibodies was significantly reduced^29^. Our results indicated that the GGGS-AD-4 protein might not mimic the entire gB and lost essential conformation to induce effective antibodies. GGGS-AD-4 alone is not an optimal vaccine design domain.

### Sortagging GGG-SP55 and GGG-SP70 to VLPs surface with high density

**Figure 4:**
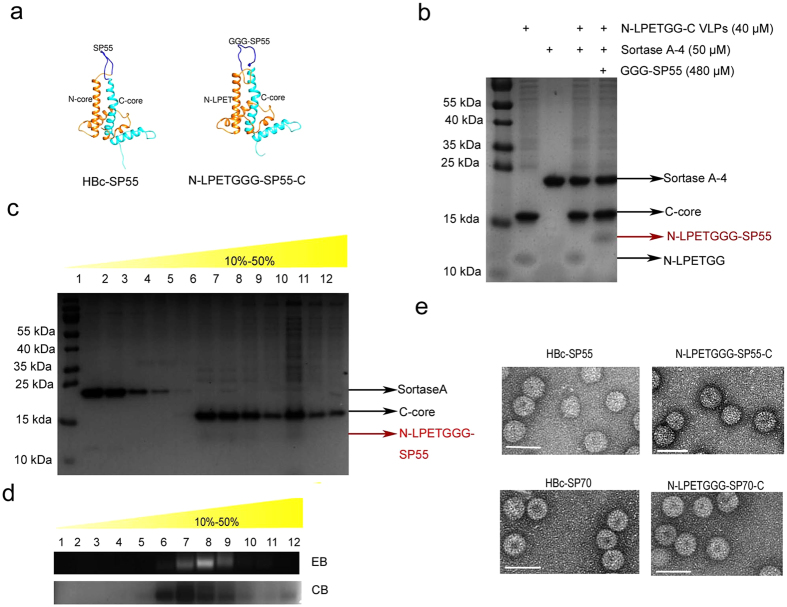
Chemoenzymatic conjugation of GGG-SP55 to N-LPETGG-C VLPs with high density. (**a**) MODELLER predicted that SP55 and GGG-SP55 would be exposed on the surface of the VLPs. The models were viewed with UCSF Chimera. N-core is orange; SP55 and GGG-SP55 are blue; and C-core is cyan. (**b**) GGG-SP55 was coupled to N-LPETGG-C VLPs with greater than 90% efficiency. (**c**) Conjugated product was separated by sucrose gradient density ultracentrifugation and analyzed by Tricine-SDS-PAGE. (**d**) NAGE assay was performed to monitor the sucrose gradient separation of reacted mixture. (**e**) TEM analysis of genetically inserted HBc-SP55, HBc-SP70, and sortagged N-LPETGGG-SP55-C and N-LPETGGG-SP70-C VLPs. White bar: 50 nm.

Structural modeling by MODELLER predicted that genetic inserted or sortagged epitopes (SP55 and SP70) were located at the c/e1 loop, and exposed on the surface of the N-LPETGG-C VLP scaffold (Fig. 4a, Supplementary Fig. 6a). To test whether the prediction was correct, we performed ELISA with SP70 monoclonal antibody^21^ and found that this antibody specifically detected HBc-SP70 and N-LPETGGG-SP70 (Supplementary Fig. 7e), which proved that the SP70 epitope was presented at the surface or the VLP and thus it was able to be detected by the assay. Like sortagging GGGS-AD-4 to N-LPETGG-C VLPs, GGG-SP55 was coupled to N-LPETGG-C VLPs with high density (Fig. 4b). From 1.5-fold to 6-fold of GGG-based eptitopes, more epitopes were coupled to VLPs after incubation of higher concentrations of antigens. No obvious increase in the target products was observed when the antigens were 12-fold compared with 6-fold (Supplementary Fig. 6b). We used 480 μM GGG-SP55 and GGG-SP70 to ensure greater coupling efficiency for the relatively low cost of synthesized peptides. The coupling efficiency was estimated at more than 90% according to the decrease in the intensity of N-LPETGG (Fig. 4b). VLPs sortagged with GGG-SP55 were separated by sucrose gradient density ultracentrifugation (Fig. 4c) and were analyzed by NAGE (Fig. 4d). VLPs tagged with GGG-SP70 were assayed in the same manner (Supplementary Fig. 6c,d). Sortagging GGG-SP70 was confirmed by western blotting assay (Supplementary Fig. 6e). About 600 μg of target products were obtained when 1 mg of N-LPETGG-C VLPs were used for reaction. N-LPETGG-C VLPs coupled with GGG-SP55 and GGG-SP70 retained the VLP structure (Fig. 4e).

### EV71 epitopes sortagged to HBc VLPs were highly immunogenic

**Figure 5:**
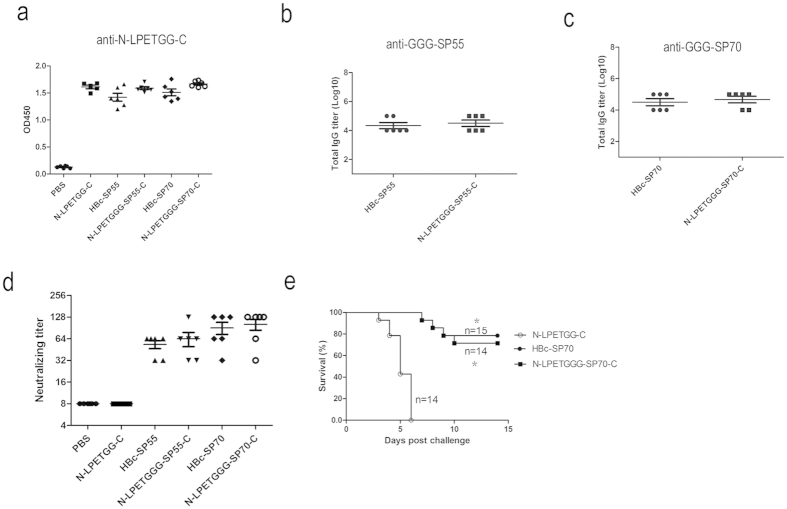
Immunization studies of mice vaccinated with PBS, N-LPETGG-C, HBc-SP55, HBc-SP70, N-LPETGGG-SP55-C, or N-LPETGGG-SP70-C. (**a**) All of the HBc-based VLPs induced an anti–N-LPETGG-C VLP IgG response with a similar level. (**b–c**) HBc-SP55/70 and N-LPETGGG-SP55/70-C elicited similar strong specific IgG response. (**d**) Injection of EV71 epitope-based HBc VLPs triggered neutralizing antibodies. Sortagged VLPs performed as well as genetically fused VLPs in neutralizing EV71 entry. (**e**) Anti-HBc-SP70 and anti-N-LPETGGG-SP70-C serums notably protected 7-day-old ICR mice from EV71 lethal challenge (*P < 0.05, by Log-rank (Mantel-Cox) test).

We then assessed the immunogenicity between VLPs sortagged with GGG-epitopes and VLPs genetically fused with epitopes by immunization study. The vaccination schedule was the same as above. No-split genetic insertion of HBc-SP55 and HBc-SP70 VLPs was prepared (Supplementary Figs 4 and 5) and injected as a positive control. All of the HBc VLPs, including split and no split, elicited an anti–N-LPETGG-C VLPs IgG response (Fig. 5a). Both HBc-SP55 and N-LPETGGG-SP55-C induced anti-GGG-SP55 IgG response (Supplementary Fig. 7a,b). No significant difference of specific IgG response was detected (Fig. 5b). Similar anti-GGG-SP70 IgG response was observed as well (Supplementary Fig. 7c,d, Fig. 5c). We then measured the neutralizing efficacy of anti-EV71 epitope IgG antidodies. Anti-SP70 serums showed higher neutralizing titers against EV71 than anti-SP55 serums. Like IgG titers, the neutralizing titers between genetic inserted groups and sortagged groups showed no apparent difference (Fig. 5d). Since SP55 and SP70 were well defined^21^, and the latter triggers higher neutralizing titer, we went on to check whether anti-SP70 serums generated by immunizing with the genetically inserted or sortagged chimeric VLPs had similar protection efficacy in an *in vivo* challenge assay. To do this, 7-day-old ICR mice were intraperitoneally injected with 100 μl anti-N-LPETGG-C, anti-HBc-SP70 or anti-N-LPETGGG-SP70-C serums^21^. They were inoculated with lethal dose of EV71 one day later and monitored for 14 days. Mice received anti-SP70 serums were remarkably protected against lethal EV71 challenge. The survival rates between the two experimental groups were similar (Fig. 5e). In general, the chimeric VLPs decorated in our approach performed as well as those modified by genetic engineering in the immunization study.

### Propose a novel chimeric VLPs design strategy

**Figure 6:**
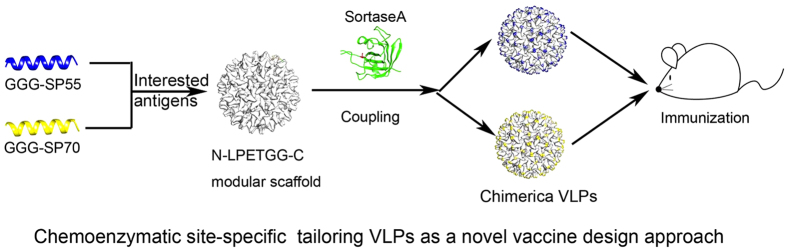
Display of interested antigens on HBc platform by sortagging. The chemically synthesized GGG-SP55 and GGG-SP70 are coupled to VLP modular scaffold by sorase A-4 mediated sortagging. Then the new chimeric VLPs are ready for immunization.

According to our results, we propose a novel chimeric VLPs design strategy (Fig. 6). Our new technique utilizes chemoenzymatic coupling to covalently link chemically synthesized peptides to the surface of the VLP scaffold. It is more convenient than constructing clones and expressing chimeric VLPs by using the genetic insertion method. Our new technique is especially helpful to display large number of epitopes, epitope library for example, on VLP platform. We showed that this reaction was reliable and replicable. Besides, the majority of VLP was occupied by interested antigens. Thus, our platform showed great potential to display a variety of interested antigens. Additionally, chemically synthesized epitopes allow other modification which is out of the range of genetic fusion. Our modular platform has the advantages of versatility equivalent to that of chemical conjugation, proper orientation, and the high density possessed by genetic insertion. Our strategy thus surpasses the traditional vaccine design approaches and represents a great advance in new VLP vaccine production.

## Discussions

VLPs are used as universal vaccine delivery system due to their intrinsic characteristic of enhancing immune responses. By genetic insertion, epitopes and proteins are displayed on VLPs with high efficiency and in proper orientation and induce strong immune responses^30^,^31^,^32^,^33^,^34^. However, it is inconvenient to display different antigens on one modular VLP platform. Chemical conjugation provides a solution to overcome this inconvenience. In clinical trials, chemical conjugated VLPs have been explored for treatment of autoimmune hypertension^35^, Alzheimer’s disease^36^, melanoma^37^, and tumor^38^, but it is still challenging to display antigens in a very specific manner. The coupling efficiency is another limit. Generally speaking, both of the traditional vaccine design approaches have pros and cons and are unsatisfactory in producing new vaccines.

In this study, we have developed a strategy that possesses the major advantages of genetic insertion and chemical conjugation. Through chemoenzymatic site-specific conjugation mediated by transpeptidase, antigens are displayed on the VLP surface in proper orientation and with high density. There is no need to activate VLPs with excess heterobifunctional cross-linker, which makes coupling a simple process. By chemoenzymatic site-specific coupling, interested molecules are more easily displayed on the VLP surface at a high density. The only drawback of our approach is the requisition of excess substrates. The excess of antigens can be reduced by modification of LPETGG to depsipeptide, which makes sortagging irreversible^39^. In addition, immobilization of sortase A on sepharose beads will decrease the sortagging cost and facilitate large-scale vaccine production^40^. Because specific IgG responses against antigens displayed on VLPs by chemoenzymatic coupling are greatly improved, our strategy also shows great potential in the promotion of antibody production. A previous study used an antibody scaffold, αDEC205, to deliver a set of antigens to dendritic cells (DCs) as a new vaccine approach^28^. This antibody scaffold elicited specific T-cell immune responses against loaded antigens. Such strategy is useful in immunotherapeutic treatment, but lacks antibody response. Compared with antibody scaffold, VLP scaffold used in our system elicits high titer anti-antigen antibodies which can be used in prophylactic vaccination. Both technologies have their own advantages and can be applied for next-generation vaccine development.

Our results demonstrate that our proposed vaccine design strategy constitutes a great advance in the bioengineering production of new vaccines. In our new coupling system, antigens are conjugated to the VLP surface in a simple, fast, site-specific, and highly efficient manner. Our new system is unique for its integration of the advantages of genetic fusion and chemical conjugation in the modification of VLPs. New vaccine production engineering will be facilitated by our strategy. In summary, interested molecules will be displayed on the VLP surface in a simple, fast, convenient, and versatile way by our proposed technology.

## Methods

### Construction of clones used in coupling system

The split N-LPETGG-C molecule contained N-LPETGG, a ribosome binding site (RBS) and C-core to form VLP structure with 1:1 N-LPETGG and C-core. A stop codon was introduced to N-LPETGG. The N-LPETGG-C molecule was constructed by overlap-PCR. The discontinuous GGGS-AD-4, aa 112–132 and 344–438 with Ile-Ala-Gly-Ser-Gly linker were cloned from cDNA of HCMV strain AD 169 by overlap-PCR. Other clones were constructed according to standard protocol of molecular cloning. All of the clones used in coupling system were inserted to pET28a vector and expressed in BL21 (DE3) plysS.

### Preparation of components used in coupling system and for immunization study

The split HBc N-LPETGG-C VLPs, HBc-SP55 and HBc-SP70, were sequentially purified by ammonium sulfate precipitation and sucrose gradient ultracentrifugation as mentioned in our previous study^26^ with slight modification. Ultracentrifugation was performed at 39,000 rpm (SW41 rotor; Beckman Optima MAX-XP ultracentrifuge) over sucrose gradient (10% to 50% w/v in 50 mM Tris [pH 7.5], 150 mM NaCl [NT buffer]) for 2 h. All of the sortagged products were recovered from reacted mixtures in the same condition. Fractions 7 to 10 were collected and dialyzed against 1 L NT buffer overnight at 4 °C. The VLPs were concentrated by a 100-kD cutoff Amicon Ultra centrifugal filter to 2 mg/ml. GGG-SP55 (gggpdsreslawqtatnp) and GGG-SP70 (gggyptfgehkqekdley) were commercially synthesized by the company Gi’er in Shanghai according to previous study^21^. Sortase A-4-6xHis and GGGS-AD-4-6xHis were purified with IMAC and then further purified by anion exchange on a fast-flow monoQ column (Pharmacia). The aggregates of sortase A-4 were further removed by sucrose gradient density ultracentrifugation. Fractions 1 to 4 were collected. The protein buffers were exchanged with NT buffer. The protein concentrations were measured with Beyotime BCA kits. The working solution for sortagging was NT buffer with 2 mM CaCl_2_. All reactions were achieved at room temperature between 20 °C and 26 °C. The reaction was terminated by sucrose gradient ultracentrifugation of sortagged products from sortase A-4 and GGG- antigens. Fractions 7-10 were collected and dialyzed against PBS. Sortagged products were concentrated to 1mg/ml by a 100-kD cutoff Amicon Ultra centrifugal filter. The target products were stable at 4 °C for a couple of weeks. For more than one year store, −80 °C condition was essential. Different batches of sortagged products for immunization study were monitored by Tricine-SDS-PAGE analysis. This reaction was highly reproducible. The sortagging efficiency was estimated by the decrease intensity of N-LPETGG band. The recovery rates were calculated by comparing the sortagged VLPs products with initial N-LPETGG-C VLPs. More than 50% recovery rates were obtained.

### Transmission electron microscopy (TEM) assay of VLPs

The VLP samples (0.1 mg/ml, 5 μl) that were negatively stained with 1% uranyl acetate were monitored with a Tecnai G2 Spirit microscope at 120 kV at 67,000 × magnification.

### Immunization studies

The 6- to 8-week-old female BALB/c mice were vaccinated with 10 μg proteins or VLPs in PBS (100 μl) with 1:1 v/v aluminium hydroxide wet gel adjuvant (Invivogen). All six mice in each group received their first injection at week 0 and boosters at weeks 2 and 4. Blood samples were taken 2 weeks after each immunization. The immunization studies were approved by the Institutional Animal Care and Use Committee at the Institut Pasteur of Shanghai. All the experiments were carried out according to approved guidelines.

### Evaluation of IgG responses

ELISA analysis and neutralization against EV71 was carried out as described before, except that the serum samples in the ELISA assay were diluted to 1:1000^21^. In brief, 50 ng of N-LPETGG-C or 100 ng antigens (GGGS-AD-4, GGG-SP55 or GGG-EV71 epitope) were applied to each well of 96-well plate. After coating over 12 h at 4 °C, the plates were washed by PBST (0.05% Tween 20 in PBS) for three times. All the following steps were washed by PBST for at least three times. Then the plates were incubated at 37 °C by 5% nonfat milk (in PBST) to block non-specific binding for 1 h. After that, the plates were added by 1:1000 diluted serum samples in PBST at 37 °C for 2 h incubation. Goat anti-mouse IgG-HRP (50 μl /well, 1:1000 in PBST) was then added for 1 h incubation at 37 °C. After TMB color development, all the plates were read at 450 nm. The ELISA data were analyzed by graphpad prism 5. The titers of IgG antibodies were measured by sequential 10-fold dilution of serums in PBS.

### Neutralizing analysis against EV71 and HCMV

The neutralizing analysis was carried out by 96-well plates. Equal volumes of 100 PFU EV71 strain G082 and 2-fold serially diluted serum samples (50 μl/well) were incubated at 37 °C for 1 h. Then 100 μl of 1.5 × 10^5^ cells/ml RD cells were added to each well. The initial dilution of the neutralizing assay was 8-fold, and the PBS and N-LPETGG-C control groups were defined as 8-fold. After incubation at 37 °C CO_2_ incubator for 3 days, the cytopathic effects (CPE) were monitored. All immunization studies of EV71 epitope-based VLPs were repeated two times. The neutralizing analysis against HCMV strain AD 169 (GFP insertion after IE2 for monitor) was done at similar condition with 800 PFU HCMV strain AD 169 and 1.5 × 10^5^ cells/ml MRC5 cells in each well. The CPE were assayed by fluorescence microscopy after 5 days post infection. No inhibitions of HCMV entry were observed. The neutralizing results were analyzed by graphpad prism 5.

### EV71 lethal challenging *in vivo* assay

To evaluate the effect of anti-EV71 epitope antibodies, we performed *in vivo* assay against EV71 in ICR mice with slight modification according to previous study^21^. The *in vivo* assay was carried out at biosafety level 2 laboratories of Institut Pasteur of Shanghai. This assay was approved by the biosafety core facility and the Institutional Animal Care and Use Committee at the Institut Pasteur of Shanghai. All the experiments were done according to approved guidelines. Three groups of 7-day-old ICR mice received intraperitoneal injection of 100 μl anti-N-LPETGG-C, anti-HBc-SP70 or anti-N-LPETGGG-SP70-C serum/mouse. The mice were intraperitoneally challenged with 2.1 × 10^4^ TCID_50_ of EV71/MAV-W (From Zhong Huang’s lab, diluted in 40 μl sterile PBS buffer) 24 h later. All mice were daily monitored for 14 days after EV71 lethal challenging. The survivals of mice were recorded.

### Statistic analysis

To evaluate the statistic difference, two-tailed student’s t-test was performed. The survival curve of EV71 lethal challenge was compared with Log-rank (Mantel-Cox) test. All the statistic analysis was done by GraphPad Prism version 5.

### Protein structure illustration

All the protein models were generated by MODULLER in UCSF Chimera. Protein sequences were added and models were automatically produced by comparing with known structure of proteins. N-LPETGG-C protein was adapted to 3D structure of HBc (1QGT)^41^. All the models were viewed by UCSF Chimera.

## Additional Information

**How to cite this article**: Tang, S. *et al.* A Modular Vaccine Development Platform Based on Sortase-Mediated Site-Specific Tagging of Antigens onto Virus-Like Particles. *Sci. Rep.* **6**, 25741; doi: 10.1038/srep25741 (2016).

## Supplementary Material

Supplementary Information
