# Supplementary material for: A Modular Vaccine Development Platform Based on Sortase-Mediated Site-Specific Tagging of Antigens onto Virus-Like Particles
Source: Sci Rep. 2016 May 12;6:25741. doi: 10.1038/srep25741 (PMC4864371; doi:10.1038/srep25741)
Supplement: Supplementary Information [file srep25741-s1.pdf]

Supplementary Document

A Modular Vaccine Development Platform Based on Sortase-Mediated Site-Specific Tagging of Antigens onto Virus-Like Particles

ShuBing Tang[1], BaoQin Xuan[1], XiaoHua Ye[2], Zhong Huang[2*] & Zhikang Qian[1*]

[1]Unit of Herpesvirus and Molecular Virology, Key Laboratory of Molecular Virology & Immunology, Institut Pasteur of Shanghai, Chinese Academy of Sciences, Shanghai 200031, China.

[2]Unit of Vaccinology and Antiviral Strategies, Key Laboratory of Molecular Virology & Immunology, Institut Pasteur of Shanghai, Chinese Academy of Sciences, Shanghai 200031, China.

[*]Correspondence authors: zkqian@ips.ac.cn (ZQ) and huangzhong@sibs.ac.cn (ZH)

SUPPLEMENTARY FIGURES

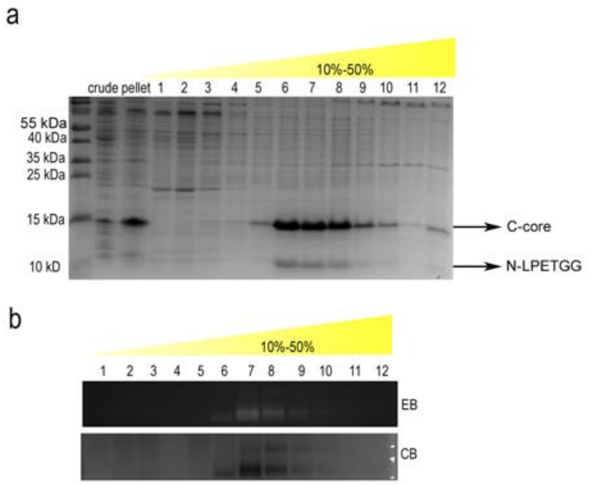

Figure 1. Ammonium sulfate precipitation enrichment and sucrose gradient separation of split N-LPETGG-C VLPs. (a) Tricine-SDS-PAGE CB staining and (b) NAGE analysis of ammonium

sulfate precipitation and sucrose gradient ultracentrifugation separation of split HBc N-LPETGG-C. Crude—supernatant of crude lysate of *E.coli*. Pellet—resuspension of ammonium sulfate–precipitated pellets of VLPs. Numbers 1 to 12 refer to different fractions of sucrose gradient ultracentrifugation. Fractions 7 through 10 were collected.

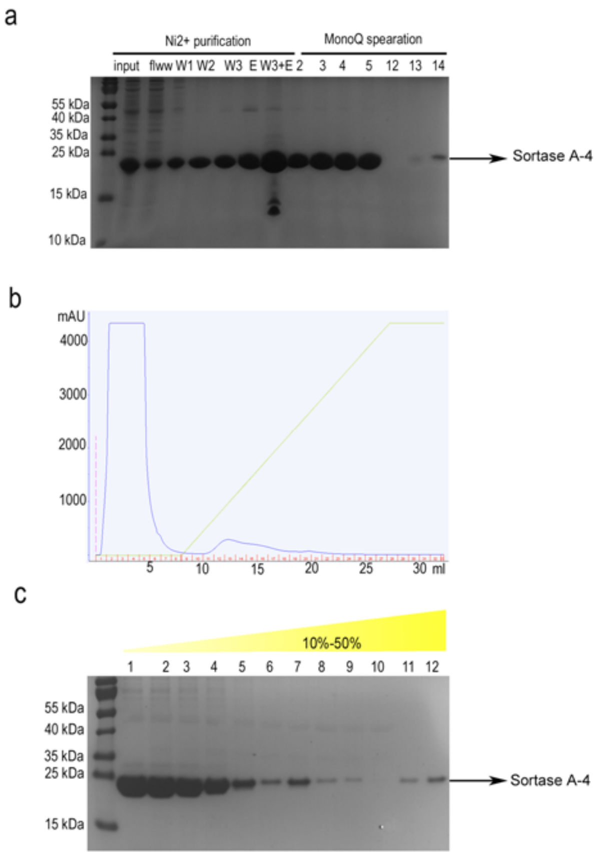

Figure 2. Immobility metal affinity chromatography (IMAC) purification, anion exchange further separation and sucrose gradient ultracentrifugation remove aggregates of sortase A-4. (a) CB analysis of IAMC and anion exchange purification of sortase A-4. Input—supernatant of the crude lysate of E.*coli*; flow—flow-through after Ni^{2+} resin binding; W1—25 mM imidazole in NT buffer wash; W2—50 mM imidazole in NT buffer wash; W3—75 mM imidazole in NT buffer wash; E—250 mM imidazole in NT buffer elution; W3+E—wash 3 and elution. The monoQ fractions of 2 to 5 were retained. (b) Uv Profile of sortase A-4 purified by monoQ. (c)

Sucrose gradient ultracentrifugation to remove the aggregates of sortase A-4. Fractions 1 to 4 were collected.

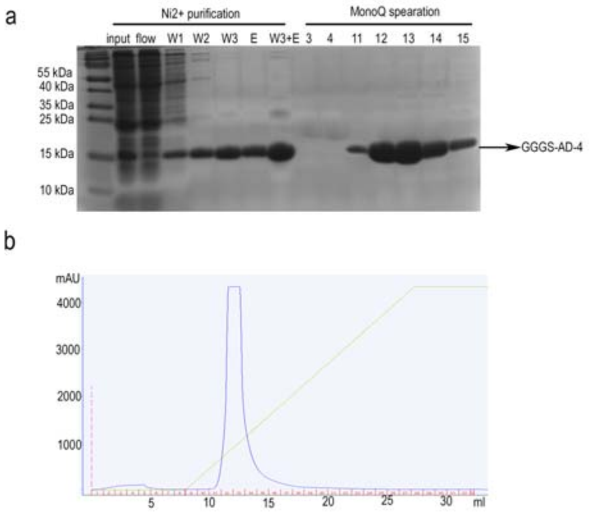

Figure 3. Immobility metal affinity chromatography (IMAC) purification and anion exchange further separation of GGGS-AD-4. (a) CB analysis of IAMC and anion exchange purification of GGGS-AD-4. Input—supernatant of the crude lysate of *E. coli*; flow—flow-through after Ni^{2+} resin binding; W1—25 mM imidazole in NT buffer wash; W2—50 mM imidazole in NT buffer wash; W3—75 mM imidazole in NT buffer wash; E—250 mM imidazole in NT buffer elution; W3+E—wash 3 and elution. Fractions 12 to 15 were collected. (b) Uv profile of GGGS-AD-4-6 purified by monoQ.

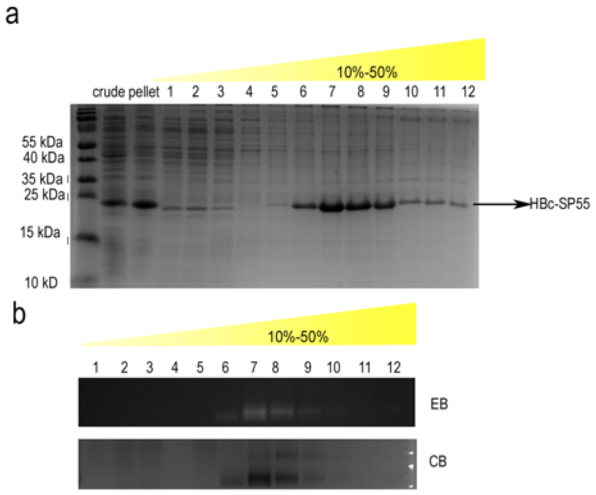

Figure 4. Ammonium sulfate precipitation enrichment and sucrose gradient separation of no-split HBc-SP55 VLPs. (a) Tricine-SDS-PAGE CB staining and (b) NAGE analysis of ammonium sulfate precipitation and sucrose gradient ultracentrifugation separation of split HBc N-LPETGG-C. Crude—supernatant of the crude lysate of E.*coli*. Pellet—resuspension of ammonium sulfate precipitated pellets of VLPs. Numbers 1 to 12 refer to different fractions of sucrose gradient ultracentrifugation. Fractions 7 to 10 were collected.

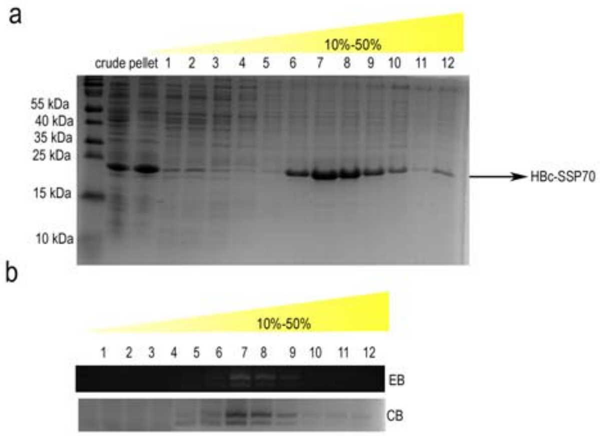

Figure 5. Ammonium sulfate precipitation enrichment and sucrose gradient separation of no-split HBc-SP70 VLPs. (a) Tricine-SDS-PAGE CB staining and (b) NAGE analysis of ammonium sulfate precipitation and sucrose gradient ultracentrifugation separation of split HBc N-LPETGG-C. Crude—supernatant of crude lysate of E.*coli*. Pellet—resuspension of ammonium sulfate precipitated pellets of VLPs. Numbers 1 to 12 refer to different fractions of sucrose gradient ultracentrifugation. Fractions 7 to 10 were collected.

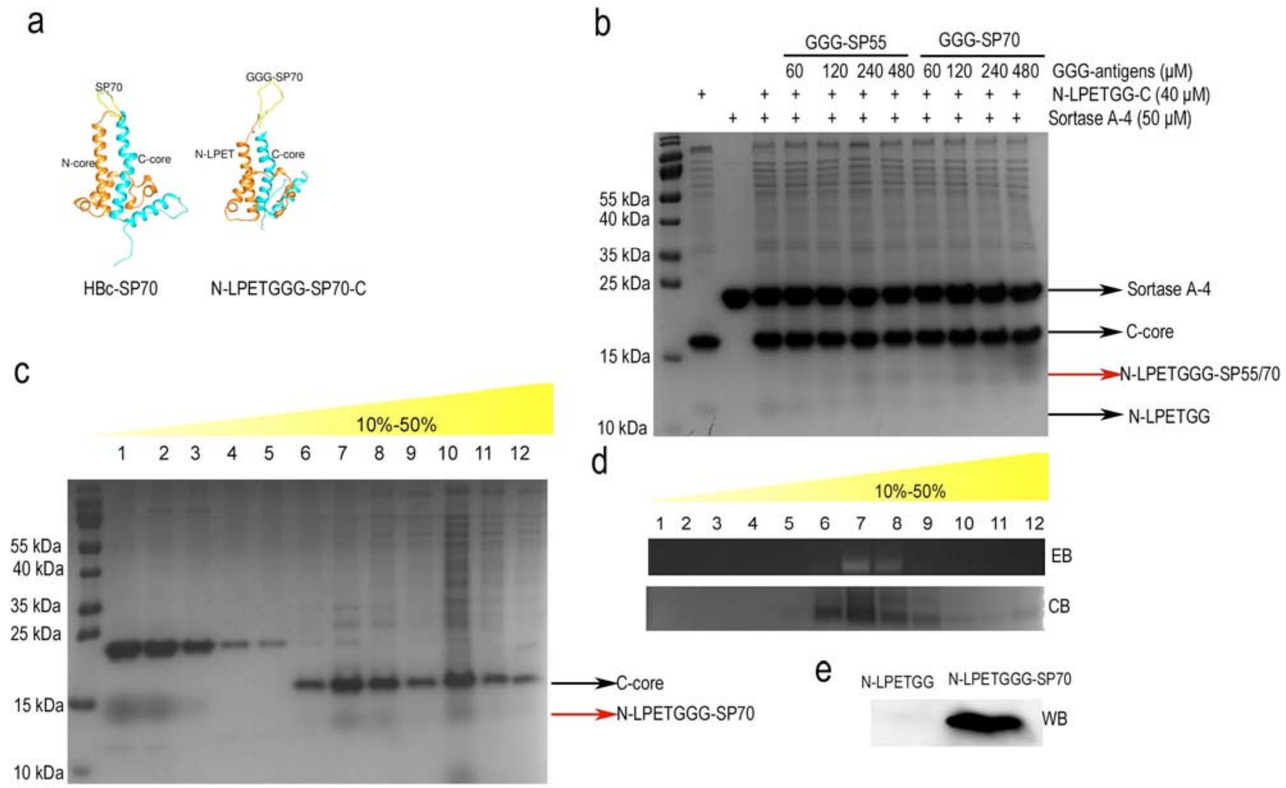

Figure 6. Coupling GGG-SP55 and GGG-SP70 to split N-LPETGG-C VLPs. (a) MODELLER predicted that SP70 and GGG-SP70 would be exposed on the surface of the VLPs. Models were viewed with UCSF Chimera. N-core is orange, SP70 and GGG-SP70 epitopes are yellow; and C-core is cyan. (b) Different concentrations of GGG-SP55 and GGG-SP70 coupled to N-LPETGG-C VLPs. (c) Tricine-SDS-PAGE and (d) NAGE analysis of sucrose gradient density ultracentrifugation separation of N-LPETGGG-SP70-C VLPs from reacted-mixture. (e) Western blotting analysis of N-LPETGG-C VLPs and N-LPETGGG-SP70-C VLPs to confirm sortagging by anti-SP70 monoclonal antibody.

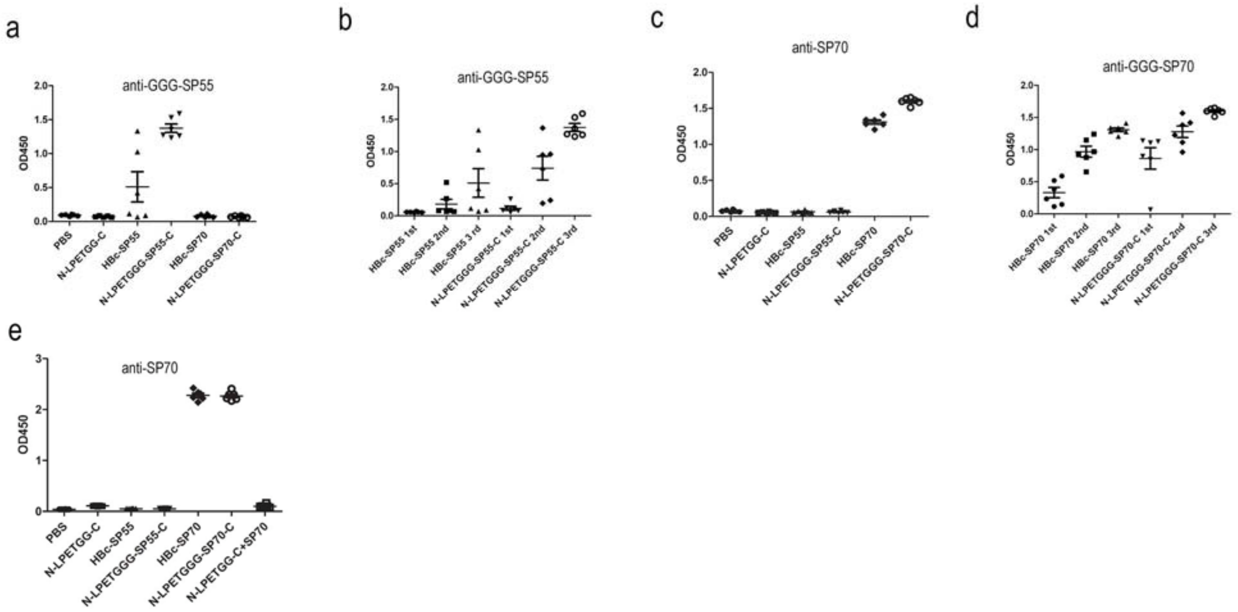

Figure 7. ELISA assay of anti-GGG-SP55, anti-GGG-SP70 and prepared chimeric VLPs. (a) The anti-GGG-SP55 IgG antibody titers were detected by ELISA. Only SP55-based VLPs elicited specific IgG response. (b) The change of anti-GGG-SP55 IgG antibody titers after three doses immunization of HBc-SP55 and N-LPETGGG-SP55-C were measured by ELISA. (c) The anti-GGG-SP70 IgG antibody titers were detected by ELISA. Only SP70-based VLPs elicited specific IgG response. (d) The change of anti-GGG-SP70 IgG antibody titers after three doses immunization of HBc-SP70 and N-LPETGGG-SP70-C were measured by ELISA. (e) Prepared chimeric VLPs were assayed by SP70 monoclonal antibody from Huang’s lab with PBS, N-LPETGG-C HBc-SP55, N-LPETGGG-SP55-C and purified VLPs VLPs fractions from reactions without sortase A-4 (named N-LPETGG-C+SP70) as control. Each sample was repeated for 6 times. Both HBc-SP70 and N-LPETGGG-SP70-C showed specific binding of SP70 monoclonal antibody. Since protein maintained nature structure during ELISA assay, our result suggested that genetically inserted SP70 and coupled GGG-SP70 were displayed on VLP surface.
